# Studying Cardiac Neural Network Dynamics: Challenges and Opportunities for Scientific Computing

**DOI:** 10.3389/fphys.2022.835761

**Published:** 2022-04-29

**Authors:** Nil Z. Gurel, Koustubh B. Sudarshan, Sharon Tam, Diana Ly, J. Andrew Armour, Guy Kember, Olujimi A. Ajijola

**Affiliations:** ^1^ UCLA Neurocardiology Research Program of Excellence, Los Angeles, CA, United States; ^2^ UCLA Cardiac Arrhythmia Center, UCLA Health System, Los Angeles, CA, United States; ^3^ Department of Engineering Mathematics and Internetworking, Dalhousie University, Halifax, NS, Canada; ^4^ UCLA Department of Bioengineering, Los Angeles, CA, United States; ^5^ Molecular, Cellular and Integrative Physiology Program, UCLA, Los Angeles, CA, United States

**Keywords:** neurocardiology, sudden cardiac death (SCD), closed-loop control, cardiac nervous system, cardiac function

## Abstract

Neural control of the heart involves continuous modulation of cardiac mechanical and electrical activity to meet the organism’s demand for blood flow. The closed-loop control scheme consists of interconnected neural networks with central and peripheral components working cooperatively with each other. These components have evolved to cooperate control of various aspects of cardiac function, which produce measurable “functional” outputs such as heart rate and blood pressure. In this review, we will outline fundamental studies probing the cardiac neural control hierarchy. We will discuss how computational methods can guide improved experimental design and be used to probe how information is processed while closed-loop control is operational. These experimental designs generate large cardio-neural datasets that require sophisticated strategies for signal processing and time series analysis, while presenting the usual large-scale computational challenges surrounding data sharing and reproducibility. These challenges provide unique opportunities for the development and validation of novel techniques to enhance understanding of mechanisms of cardiac pathologies required for clinical implementation.

## Introduction

Beat-to-beat control of cardiac function requires adaptive adjustments of cardiac electromechanical activity to meet the organism’s blood flow needs. The closed-loop cardiac control network hierarchy consists of the intrinsic cardiac nervous system, the sympathetic and parasympathetic arms of the autonomic nervous system, peripheral ganglia, spinal cord, brain stem, and higher centers in the central nervous system (Ardell et al., 2016; Shivkumar et al., 2016). Contrary to the viewpoint where the peripheral nervous system functions as a conduit for centrally-derived inputs (Yuste, 2015), neural control of cardiac function involves a hierarchy of interconnected neural networks that regionally control indices such as heart rate, blood pressure, or respiration (Ardell et al., 2016; Shivkumar et al., 2016).

Substantial experimental and clinical work has focused on neural contributions to heart rate, heart rate variability, and blood pressure anomalies and associated pathologies including heart failure, myocardial infarction, coronary artery disease, and hypertension (Sessa et al., 2018; Pan et al., 2020). At a population level, elevated resting heart rate and blood pressure, reduced heart rate variability, and depressed baroreflex sensitivity correlate with increased risks of cardiovascular disease, mortality, arrhythmia, and negative health outcomes (Jones et al., 2008; Zhang et al., 2016; Grassi et al., 2019; Fuchs and Whelton, 2020). While these biomarkers remain relevant at a population level, their usefulness to assess risk of adverse outcomes for individual patients is limited due to regionality of the control hierarchy (Huikuri and Stein, 2013; Kember et al., 2013; Ajijola, 2016; Mitrani and Myerburg, 2016; Pan et al., 2020).

The cardiac neural control network may be characterized as a closed-loop control system with the central nervous system as one component (Armour, 2004; Leenen, 2007; Hirooka, 2010; Scherbakov and Doehner, 2018). At the peripheral level of the control hierarchy, afferent and efferent activities arise from locally interconnected feedback loops of ganglia and interconnecting neurons (Ardell et al., 2016; Shivkumar et al., 2016). While the peripheral and central levels are in constant communication, the peripheral level is independently capable of maintaining basic cardiac function (Ardell et al., 1991; Smith et al., 2001a; Smith et al., 2001b). An important consequence is that autonomic control and the heart may become compromised while continuing to maintain function as indicated by measures such as heart rate and blood pressure (Armour, 2004; Kember et al., 2013).

**FIGURE 1:**
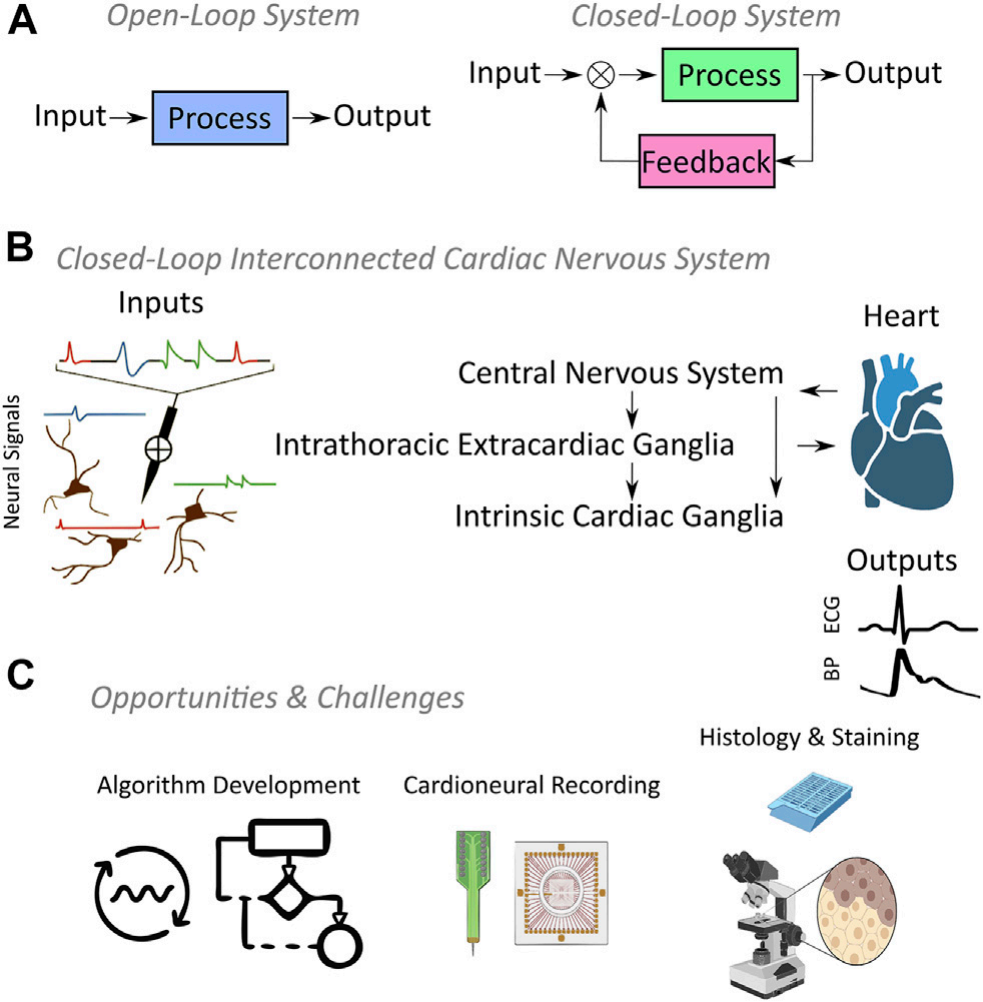
**(A)** Fundamental difference between open-loop and closed-loop systems is that the system output is regulated by feedback in closed-loop systems, while open-loop systems have no feedback. **(B)** Cardiac nervous system represents a three-tier closed-loop control hierarchy where each tier exhibits neural inputs resulting in functional outputs such as the electrocardiography (ECG) or blood pressure (BP). **(C)** Open source algorithm development and novel cardioneural recording technologies supported by histologic studies will propel the field of neurocardiology forward.

Substantial progress has been made in the description of network components down to the cellular and genomic levels (Moss et al., 2021; Rajendran et al., 2019; Hanna et al., 2021), but the principles and mechanisms governing higher level function and neuro-mechanical linkages are not well defined. This is partly due to the closed-loop nature across levels of the cardiac control hierarchy: it has no simple open-loop analogue to provide a direct linkage between neural activity and functional targets (Figure 1). A useful strategy is to de-link levels within the control hierarchy, and this has been successfully used in experimental designs to gain insight into neural contributions to cardiac function and in clinical interventions as a last resort (Ardell et al., 1991; Smith et al., 2001a; Smith et al., 2001b). A main requirement is that experimental methods must directly measure integration within the cardiac neural control system and the linkage of this system to control targets while in closed-loop operation.

In this review, we will outline the fundamental studies focusing on closed-loop information processing within cardiac neural hierarchy. Our emphasis will be less on past achievements and more on identifying trends that are shaping the field. We will discuss how computational methods can help to guide improved experimental designs, quantify the value of small datasets, and become useful for attacking long-standing problems that span multiple scales in space and time. The size and complexity of these next generation cardio-neural datasets require sophisticated strategies for signal processing, time series analysis, and dimensionality reduction, while presenting the usual large-scale computational challenges surrounding data sharing and reproducibility. These challenges provide unique opportunities to further technological development and computational pipelines to drive improved understanding of mechanisms of cardiac pathologies.

## Neural Recording Literature in Cardiac Nervous System

The cardiac nervous system offers untapped opportunities to understand mechanisms of cardiac disease and develop novel therapeutic strategies. Manipulation of the cardiac nervous system is a promising approach to mitigate the onset and progression of cardiac pathology. However, its implementation requires an understanding of the neural-mechanical linkages if safe and effective therapeutic strategies are to be developed. In Table 1, a representative selection of the research literature into neuro-mechanical linkages spanning the late 1960s to 2021 is provided. Earlier studies include research into anatomy and function of the stellate ganglion, right atrial ganglionated plexus, spinal cord, and nodose with single-unit recordings (Ja¨nig and Szulczyk, 1980; Armour, 1983; Armour, 1985; Armour, 1986; Gagliardi et al., 1988; Boczek-Funcke et al., 1992; Boczek-Funcke et al., 1993; Armour et al., 1998; Ardell et al., 2009; Salavatian et al., 2019a; Foreman et al., 2015; Foreman and Qin, 2009). With the advent of improved recording methods these approaches have evolved to more recent studies involving multi-unit recordings. Earlier computational methods utilized single neuron recordings and the phase relationship of a neuron’s activity to common cardiac measures were considered. Functional properties of neurons were examined through the neural response to a variety of stimuli such as rhythmicity of the neural firing pattern to functional recordings such as blood pressure and respiration, mechanical touch, electrical stimulation, and pharmacological agents.

**TABLE 1:** Research literature probing into cardiac nervous system, listed as regions, recording type (single/multi unit), anesthetic agent, studied species, computational methods used, number of recording electrodes, electrode type, and software used for analysis. The reference numbers correspond to the references in the manuscript.

Ref #	Year	Region	Recording	Anesthesia	Species	Methods used	# Electrodes	Electrode type	Software
Foreman et al. (1975)	1975	spinal cord	single unit	halothane, alpha chloralose	monkey	firing rate, conduction velocity	1	platinum wire	N/A
Ja¨nig and Szulczyk, (1980)	1980	lumbar preganglionic neurons	single unit	ketamine hydrochloride, alpha chloralose	cat	firing rate, conduction velocity	1	platinum wire	N/A
Blair et al. (1981)	1981	spinal cord	single unit	ketamine, alpha chloralose	monkey	firing rate	1	stainless steel	N/A
Blair et al. (1982)	1982	spinal cord	single unit	ketamine, alpha chloralose	monkey	firing rate, conduction velocity	1	tungsten, class	N/A
Armour, (1983)	1983	stellate ganglion	single unit	sodium pentobarbital, alpha chloralose	dog	N/A	1	N/A	N/A
Ammons et al. (1983a)	1983	spinal cord	single unit	ketamine, alpha chloralose	monkey	firing rate	1	stainless steel	N/A
Ammons et al. (1983b)	1983	spinal cord	single unit	ketamine, alpha chloralose	monkey	firing rate, conduction velocity	1	stainless steel	N/A
Ammons et al. (1984a)	1984	spinal cord	single unit	ketamine, alpha chloralose	monkey	firing rate	1	stainless steel	N/A
Ammons et al. (1984b)	1984	spinal cord	single unit	ketamine, alpha chloralose	monkey	firing rate, conduction velocity	1	stainless steel	N/A
Ammons and Foreman, (1984)	1984	spinal cord	single unit	ketamine, alpha chloralose	cat	firing rate	1	carbon tipped glass	N/A
Blair et al. (1984b)	1984	spinal cord	single unit	ketamine, alpha chloralose	monkey and cat	firing rate	1	tungsten or glass	N/A
Blair et al. (1984a)	1984	spinal cord	single unit	ketamine, alpha chloralose	cat	firing rate	1	tungsten or glass	N/A
Foreman et al. (1984)	1984	spinal cord	single unit	ketamine, alpha chloralose	cat	firing rate, stimulation latency	1	stainless steel	N/A
Ammons et al. (1985b)	1985	spinal cord	single unit	ketamine, alpha chloralose	monkey	firing rate	1	stainless steel	N/A
Ammons et al. (1985a)	1985	spinal cord	single unit	ketamine, alpha chloralose	monkey	firing rate, stimulation latency	1	N/A	N/A
Armour, (1985)	1985	middle cervical ganglion	single unit	Fentanyl citrate, alpha chloralose	dog	firing rate, action potential discharge pattern, duration, SNR	1	tungsten	N/A
Armour, (1986)	1986	stellate ganglion	single unit	alpha chloralose	dog	firing rate, firing pattern, cardiac/respiration rhythmicity	1	tungsten	N/A
Brennan et al. (1987)	1987	spinal cord	single unit	ketamine, alpha chloralose	monkey	firing rate, stimulation latency	1	stainless steel	N/A
Girardot et al. (1987)	1987	spinal cord	single unit	ketamine, alpha chloralose	monkey	firing rate, stimulation latency	1	carbon-filament glass	N/A
Gagliardi et al. (1988)	1988	right atrial ganglionated plexus	single unit	Fentanyl citrate, alpha chloralose	dog	firing rate, firing pattern, cardiac/respiration rhythmicity	1	tungsten	N/A
Bolser et al. (1989)	1989	spinal cord	single unit	ketamine, alpha chloralose	cat	firing rate, stimulation latency	1	platinum-iridium	N/A
Hobbs et al. (1989)	1989	spinal cord	single unit	ketamine, alpha chloralose	monkey	firing rate, stimulation latency	1	stainless steel	N/A
Chandler et al. (1991)	1991	spinal cord	single unit	ketamine, alpha chloralose	monkey	firing rate, stimulation latency	1	carbon-filament	N/A
Boczek-Funcke et al. (1992)	1992	thoracic sympathetic neurons	single unit	ketamine hydrochloride, alpha chloralose	cat	firing rate, firing pattern, cardiac/respiration rhythmicity	1	platinum wire electrodes	N/A
Boczek-Funcke et al. (1993)	1993	thoracic preganglionic neurons	single unit	ketamine hydrochloride, alpha chloralose	cat	firing rate, firing pattern, axonal conduction velocity, spontaneous activity, segmental location in spinal cord	1	platinum wire electrodes	N/A
Zhang et al. (1997)	1997	spinal cord	single unit	sodium pentobarbital	rat	firing rate, spikes per stimulus intensity	1	carbon-filament glass	N/A
Armour et al. (1998)	1998	left middle cervical and left stellate ganglion	single unit	thiopental sodium, alpha chloralose	dog	firing pattern, cross correlation (coherence), firing pattern	1	tungsten	N/A
Chandler et al. (2000)	2000	spinal cord	single unit	ketamine, alpha chloralose	monkey	firing rate, stimulation latency	1	carbon-filament glass	N/A
Qin et al. (2001)	2001	spinal cord	single unit	sodium pentobarbital	rat	firing rate, stimulation latency	1	carbon-filament glass	N/A
Chandler et al. (2002)	2002	spinal cord	single unit	ketamine, alpha chloralose	monkey	firing rate, stimulation latency	1	carbon-filament glass	N/A
Zhang et al. (2003)	2003	spinal cord	single unit	sodium pentobarbital	rat	firing rate, stimulation latency	1	carbon-filament glass	N/A
Qin et al. (2003a)	2003	spinal cord	single unit	sodium pentobarbital	rat	firing rate, stimulation latency	1	carbon-filament glass	N/A
Qin et al. (2003b)	2003	spinal cord	single unit	sodium pentobarbital	rat	firing rate, stimulation latency	1	carbon-filament glass	N/A
Qin et al. (2003c)	2003	spinal cord	single unit	sodium pentobarbital	rat	firing rate, stimulation latency	1	carbon-filament glass	N/A
Qin et al. (2004c)	2004	spinal cord	single unit	sodium pentobarbital	rat	firing rate, stimulation latency	1	carbon-filament glass	N/A
Qin et al. (2004a)	2004	spinal cord	single unit	sodium pentobarbital	rat	firing rate, stimulation latency	1	carbon-filament glass	N/A
Qin et al. (2004b)	2004	spinal cord	single unit	sodium pentobarbital	rat	firing rate, stimulation latency	1	carbon-filament glass	N/A
Qin et al. (2006)	2006	spinal cord	single unit	sodium pentobarbital	rat	firing rate, stimulation latency	1	carbon-filament glass	N/A
Qin et al. (2007)	2007	spinal cord	single unit	sodium pentobarbital	rat	firing rate, stimulation latency	1	carbon-filament glass	N/A
Qin et al. (2008)	2008	spinal cord	single unit	sodium pentobarbital	rat	firing rate, stimulation latency	1	carbon-filament glass	N/A
Ardell et al. (2009)	2009	middle cervical ganglion	single unit	thiopental sodium, alpha chloralose	dog	firing pattern	1	tungsten	Spike2
Goodman-Keiser et al. (2010)	2010	spinal cord	single unit	sodium pentobarbital	rat	firing rate, stimulation latency	1	carbon-filament glass	N/A
Qin et al. (2010)	2010	spinal cord	single unit	sodium pentobarbital	rat	firing rate, stimulation latency	1	carbon-filament glass	N/A
Little et al. (2011)	2011	spinal cord	single unit	sodium pentobarbital	rat	firing rate, stimulation latency	1	carbon-filament glass	N/A
Beaumont et al. (2013)	2013	right atrial ganglionated plexus	multi unit	isoflurane	dog	template matching, principal component analysis, spike rate, conditional probability	16	platinum-iridium	Spike2
Salavatian et al. (2019a)	2019	spinal cord	single unit	isoflurone, alpha chloralose	dog	spike sorting feature of software (not specified)	1	tungsten	Spike2
Dale et al. (2020)	2020	spinal cord	multi unit	inhaled isoflurane, fentanyl, alpha chloralose	pig	firing rate, cross correlation, conditional probability	64	platinum-iridium	Spike2
Yoshie et al. (2020)	2020	stellate ganglion	multi unit	inhaled isoflurane	pig	firing rate	N/A	N/A	N/A
Omura et al. (2021)	2021	spinal cord	multi unit	inhaled isoflurane, fentanyl, alpha chloralose, bupivacaine	pig	firing rate	64	platinum-iridium	iScalDyn
Salavatian et al. (2019b)	2021	nodose	multi unit	isoflurane, alpha chloralose	pig	firing rate	16	platinum-iridium	Spike2, MATLAB
Sudarshan et al. (2021)	2021	stellate ganglion	multi unit	isoflurane, chloralose	pig	unsupervised spike detection, spike rate	16	platinum-iridium	Open source, Python

Foreman et al. (1975), Foreman et al. (1984) was the first to investigate spinal cord neurons in monkeys using single-unit platinum wire electrodes through laminectomy. These efforts were followed by Blair et al. (1981), Blair et al. (1982), Blair et al. (1984a), Blair et al. (1984b), Ammons et al. (1983a), Ammons et al. (1983b), Ammons et al. (1984a), Ammons and Foreman (1984), Ammons et al. (1984b), Ammons et al. (1985a), Ammons et al. (1985b), Brennan et al. (1987), Girardot et al. (1987), Bolser et al. (1989), Hobbs et al. (1989), and Chandler et al. (1991) in cats and monkeys using tungsten, stainless steel, platinum-iridium, and carbon-filament glass electrodes. The effects of cardiovascular stressors, noxious stressors, vagal afferent stimulation, and pharmacological agents on T1-T5 spinal, spinothalamic, and spinoreticular neurons were studied in separate investigations. These studies laid the groundwork to understand the mechanisms of cardiac pain, roles of neurotransmitters, and multi-organ architecture of spinal neurons (Foreman et al., 2015). Another set of studies focused on C1-C2 spinal neurons (Zhang et al., 1997; Chandler et al., 2000; Qin et al., 2001; Chandler et al., 2002; Qin et al., 2003a; Qin et al., 2003b; Zhang et al., 2003; Qin et al., 2004a; Qin et al., 2004b), characterization of thoracic spinal neurons receiving inputs from the heart and lower airways (Qin et al., 2003c; Qin et al., 2004c; Qin et al., 2006; Qin et al., 2007; Qin et al., 2008), and multi-organ processing of cardiac nociception (Goodman-Keiser et al., 2010; Qin et al., 2010; Little et al., 2011).

Using similar electrode technologies and methods, peripheral investigations were carried out by other groups. Ja¨nig and Szulczyk (1980) investigated the functional properties of lumbar preganglionic sympathetic neurons in cats using single-unit platinum wire electrodes. The functional properties of the neurons were classified according to cardiac rhythmicity, reactions to different stimuli, and axon conduction velocity. Armour (1983) performed a set of experiments to study synaptic transmission in middle cervical and stellate ganglia in dogs after thoracic autonomic ganglia decentralization. Compound action potential shapes were studied based on their response to a number of pharmacological agents and electrical stimulation of an afferent cardiopulmonary nerve. In subsequent studies, extracellular neural activity of middle cervical and stellate ganglia neurons was recorded in dogs (Armour, 1985; Armour, 1986). Action potentials were identified based on pre-determined signal to noise ratios, action potential duration, action potential discharge pattern, and firing rates have been quantified. Neural classifications were performed based on cardiac cycle rhythmicity, respiration, respiration rhythmicity, response to mechanical distortion of the superior vena cava, heart, thoracic aorta, thoracic wall, neck, or foreleg skin, and response to stimulation of sympathetic and/or cardiopulmonary nerves. Gagliardi et al. (1988) similarly studied right atrial ganglionated plexus neural activity in dogs by finding neurons that showed cardiac rhythmicity, respiratory rhythmicity, and responded to mechanical stimuli.

Boczek-Funcke et al. (1992) split nerve bundles into fine filaments upon perineurium incision and utilized 167 single-unit platinum wire electrodes to classify 167 single preganglionic neurons in cats, based on three reflex criteria: cardiac rhythmicity (Group 1 neurons), response to noxious stimulation of the skin (Group 2 neurons), and the coupling of neural activity to central inspiratory drive (phrenic nerve activity, Group 3 neurons). Neurons that showed lack of cardiac rhythmicity but excitability to noxious skin stimuli were labelled Group 4 neurons. A subsequent work by the same group tested whether these four, functionally distinct groups differed in the distribution of their segmental origin within the spinal cord, spontaneous activity, and axonal conduction velocity (Boczek-Funcke et al., 1993). It was reported that neurons showing different reflex patterns differed in segmental location and axonal conduction velocity. A similar stimuli-response approach was undertaken to evaluate the differential selectivity of neurons in middle cervical or stellate ganglia versus intrinsic cardiac ganglia in dogs (Armour et al., 1998). The evaluated interventions were: temporary discontinuation of respiration, alteration of respiratory rate, inferior vena cava occlusion, aortic occlusion, pharmacological agent infusion, epicardial touch, and carotid sinus stimulation. Firing patterns and cross correlation of neural firing across ganglia showed similarity and dissimilarity in reflex patterns to a wide range of stimuli. Lastly, more recent multi-probe recordings of neurons used software (Spike 2, Cambridge Electronic Design) to filter and analyze middle cervical ganglion (Ardell et al., 2009) and dorsal root ganglion (Salavatian et al., 2019a) neural recordings. The totality of evidence led to the conclusion of a thoracic nervous system acting as a distributive processor with redundant cardio-regulatory control mechanisms exerted through multiple nested feedback loops.

With the advent of linear and multi-grid microelectrode arrays, it became feasible to evaluate neural activities within and between neurons. Beaumont et al. reported activity from multiple intrinsic cardiac neurons in the right atrial ganglionated plexus in dogs using a 16-channel linear array (Beaumont et al., 2013). Following an artifact removal process based on right atrial electrogram and stimulator signals, neural activities were compared in different time windows before/after interventions by the computation of the firing rate evolution (Gagliardi et al., 1988). A Skellam distribution (Hyun-Chool Shin et al., 2010) was employed to evaluate the differences in firing rate for each intervention while differential neural to stressors were evaluated via conditional probability and a chi-squared analysis was used to compare the response characteristics of the identified intrinsic cardiac neurons. Nodose and stellate ganglia neural activity were also recorded in a separate study using 16-channel microelectrode arrays (Salavatian et al., 2019b; Yoshie et al., 2020). Spike sorting was performed via principal component analysis and k-means clustering analysis. Afterwards, individual neural activity time series were extracted to study temporal profile, calculate firing rates, and quantify firing patterns with respect to applied cardiac stressor times.

In more recent studies, neural recording technology has used 64-channel neural data distributed over eight “shanks” using penetrating high-density microarrays (Dale et al., 2020; Omura et al., 2021). These studies involved recording from the thoracic spinal cord which serves to integrate cardiac control through intraspinal reflexes. Apparent from Table 1, single-unit spinal cord recordings could pinpoint to a limited set of questions at a single experiment (Foreman et al., 2015), making knowledge transfer between expensive studies challenging. High-density multi-shank recordings are attractive for spinal cord research as these regions include multi-function neural populations and making it difficult to pinpoint neural activity related to cardiac control. Dale et al. (2020) used multi-shank recordings along with a number of stressors to reveal dorsal horn and sympathetic preganglionic cardiac neurons in a pig model. Principal component analysis yielded a total of 1760 identified spinal neurons, and T2 paravertebral ganglion stimulation was used to identify/activate cardiac sympathetic preganglionic neurons. Firing rate and correlation analyses were performed for neuron identification, and percentages of neurons responding to one or more stimuli were reported. Recently, a similar experimental setup and computational methods were used to study the effects of spinal anesthesia (bupivacaine) on spinal network interactions (Omura et al., 2021). Cardiac spinal neurons were identified based on their response to a wide range of interventions and bupivacaine was reported to have cardioprotective effects as it attenuates short-term coordination between local afferent-effect cardiac neurons in spinal cord.

## Spike Detection and Spike Sorting

Studies to date have utilized event-based analyses or snapshots of experimental data, which represent only 10% of the experimental data. Analysis of short duration event regions was possible with the use of semi-automated methods with conclusions limited to static analyses. However, development of an understanding of network interactions and space-time dynamics requires the continuous analysis of entire recordings separated into baseline and event epochs. This necessitates an order of magnitude increase in processing and has driven the construction of unsupervised spike detection and classification algorithms of large-scale datasets.

Lewicki (1998) provided the earliest exploration of the techniques and challenges encountered in spike detection and sorting from extracellular microelectrode recordings. Early spike detection was achieved via window discrimination and procedures are detailed for spike sorting based on principal component analysis and component clustering. More recent reviews (Rey et al., 2015; Lefebvre et al., 2016; Hennig et al., 2019) address common challenges and techniques that are moving closer to unsupervised algorithms needed for continuous analysis of large datasets. The algorithms are necessarily tailored to the context of specific applications and measurement equipment that present disparate features.

Our recent application is characterized by ensemble neural activity where individual neurons exhibit firing rates on the order of 1 Hz without bursting (Sudarshan et al., 2021). Neural activity detected farther from the multi-channel probe represents the superposition of attenuated activity. The superposition and attenuation eventually produce recorded signals that where individual action potentials cannot be recognized, and this is termed the “noise floor.” A primary goal in analyzing multi-channel recordings is to assess network function and this is made possible by working close to the noise floor and increasing the number of recorded spikes through several orders of magnitude. We use an unsupervised approach where spikes are detected at iterated thresholds based on a competition between the number of positive and negative spikes detected at each iteration (Sudarshan et al., 2021). Regions containing spikes detected within an iteration are masked and rendered undetectable at later iterations. This approach allowed for detailed assessment of specificity over space and time of stellate ganglion population activity to specific cycles in cardiac and pulmonary dynamics during an experiment.

Following the detection of spikes, a typical spike sorting procedure involves extracting features from detected spikes and assigning them to unique clusters where each cluster would ideally represent activity from a single neuron. Rey et al. (2015) and Lefebvre et al. (2016) outlined procedures such as projection on basis functions, principal component analysis and wavelet analysis that are commonly used for feature extraction prior to clustering from detected spikes. Various clustering algorithms such as Gaussian mixtures, k-means, and density-based clustering were reviewed along with a template matching procedure for extracting activity from single neurons from the clusters. The major challenge in using these clustering methods to isolate the activity of specific neurons within a population remains the lack of an independent means to assess cluster validity. This problem is reviewed in (Foreman et al., 1975) with respect to validating spike sorting clusters with and without human-based or synthetic ground truth validations of sorted spikes. It is also explored in (Magland et al., 2020) where automated spike sorting pipelines are built for the low-dimensional spike sorting problem and compared to other approaches.

## Study Design and Data Analysis Considerations

In this section, we address experimental issues that should be considered when designing and analyzing neural recording studies to probe the cardiac nervous system and a hierarchical closed-loop controller. Table 1 lists these details for cardiac literature discussed previously.

### Single-Unit vs Multi-Unit Recordings

The range of electrode technologies has also greatly diversified the type of collected neural signals. Single-unit tungsten or platinum wire single-unit electrodes have dominated the field until 2000s (Ja¨nig and Szulczyk, 1980; Armour, 1985; Boczek-Funcke et al., 1992; Armour et al., 1998). In recent years, multi-unit or multi-channel recordings have appeared in studies due to the availability of recording technologies (Beaumont et al., 2013; Dale et al., 2020; Sudarshan et al., 2021). Both single- and multi-unit electrodes have strengths and weaknesses depending on the experimental goals.

For single-unit recordings, the target neurons must be isolated and recording electrodes should be fine-tipped with low-impedance conductors for high quality recording. Single-unit recordings may record several isolated neurons with wire electrodes in separate nerve bundles (Boczek-Funcke et al., 1992). While large electrode arrays increase the amount of collected information per unit time, they may not provide sufficient isolation. The multi-unit signals involve recording of closest *neural populations*, rather than the closest single neuron. In the recent neuroscience literature, a shift in the experimental focus to interactions of neural populations and their ensemble behaviors (Yuste, 2015; Zamani Esfahlani et al., 2020) has led to the nearly exclusive use of multi-unit recordings.

Study reproducibility requires reporting details of electrode design, statistical analyses should consider the independence of data and the addition of electrode/neuron identity as covariates. Data collected from a multi-unit electrode array have more stringent methodological constraints compared to single-unit data analysis. Multi-unit recordings cannot be classified as independent if unit isolation was unassessed whereas multiple single-unit recordings may be considered independent datasets assuming isolated neurons are being recorded.

### Reliance on Animal Models and Anesthesia

Open heart surgeries conducted in cardiac nervous system research studies require the use of anesthetic agents, a list of agents have been listed in Table 1. In large animal models such as dogs and pigs, isoflurane inhalation followed by alpha-chloralose have been dominantly employed. Ideally, the agent should not restrict the scientific interpretation while providing stable experimental conditions showing an absence of depression of cardiovascular or autonomic activity which is a disadvantage in cardiac neural recordings.

The use of open chest preparations along with the application of anesthesia in terminal animal experiments inevitably biases both study results, interpretations, and any potential extension to humans. Yet, when experiments are tightly controlled, chronic animal model studies have been proven informative to study the nature of interactions among neural populations and their evolution from normal to pathological states. Collection of neural data from human cardiac nervous system is a more difficult and constrained task as the experiment with humans cannot be regarded as terminal. Translational failure may be explained by methodological flaws and inadequate data in animal studies. To avoid translational failure, publications should clearly indicate the study details. Systematic reviews and meta-analyses play substantial role in the selection of the most promising interpretations that could be extended to humans.

### Sample Sizes and Statistical Power

There has been considerable concern surrounding reproducibility of small biomedical research datasets and contamination of literature with false positive reports due to publication pressure and lack of venues that encourage publication of negative results (Ioannidis, 2005; Button et al., 2013). This might be partly due to lack of planning in experimental design and reliance on the analysis of smaller studies compared to large clinical trials that involve dedicated personnel and a more thorough analysis. It is essential for investigators to describe number of animals, specifics of neural recording channels, neural type where relevant, along with power analyses for statistical significance and effect size for practical relevance. Effect size, a standardized measure that quantifies the size of difference or association between two groups, should be provided in addition to statistical significances to facilitate meta-analysis and reproducibility (Sullivan and Feinn, 2012).

### Data and Code Sharing

Combined resources surpass the capacity of individual research laboratories or institutional efforts (Ascoli et al., 2017). To date, some effort has been made to enable data reusability such as NeuroMorpho.Org (Ascoli et al., 2007), Neurodata Without Borders (Teeters et al., 2015), and PhysioNet (Goldberger et al., 2000). An instrumental effort within the context of cardiac nervous system has been the National Institutes of Health (NIH) Common Fund’s Stimulating Peripheral Activity to Relieve Conditions (SPARC) platform that encourages raw data sharing with proper labeling and a listing of computational methods/models (Osanlouy et al., 2021). In addition, computational techniques such as signal processing, machine learning, statistical analyses are central in data analysis and interpretation of results. Methods sections of research papers outline essential processing flows and mathematic/statistical information, but the complete linkage between raw data and the published results requires access to small scripts for statistical manipulations and to much larger routines used to process the raw data to a useable form. Public access to research codes, complete data pipelines used to construct all results is necessary for reproducibility, transparency of data/analysis assumptions, and the further development of software (Barnes, 2010; Ince et al., 2012).

## Concluding Remarks

We are in an exciting period of study of the cardiac nervous system with the availability of high-density recording technologies and advances in open-source computational pipelines and data-analytic methods suitable for closed-loop systems. The neuroscience literature offers a wide range of novel analytical tools and interventions mostly related to open-loop brain recording studies. Such experimental designs have separated inputs and outputs (Yuste, 2015), which do not extend to cardiac studies where heart in open-loop mode would have no afferent feedback and is not experimentally realizable or meaningful. The presence of afferent signals in closed-loop mode implies that efferent cardiac inputs are returned via the afferent pathway from the heart and further affects the efferent input to the heart. In this sense, inputs and outputs are unseparated and this has necessitated the development of metrics suitable for the analysis of the dynamical state of closed-loop networked control.

The requirement to analyze continuous recordings instead of focusing on stimulus-evoked regions is driving the development of unsupervised algorithms for spike detection and classification due to a large increase in data. These analyses are leading to the discovery a highly nuanced interpretation of the neural network status in normal versus diseased states that is unavailable from event-based analyses.

Moreover, reproducibility requirements are more difficult to meet for multi-unit experimental designs where changes in probe placement, animal’s autonomic status, surgical preparation, experimenter abilities, and genetic differences will lead to greater variability in experimental results. Designing elegant investigations that meet these reproducibility constraints, data, and method sharing supported by histological studies giving improved anatomical information are all required to further develop neurocardiology and improve clinical interventions.
